# Anesthetic Management of Multiple Endocrine Neoplasia Type 2A (MEN2A) Syndrome With Contralateral Recurrent Pheochromocytoma: A Case Report

**DOI:** 10.7759/cureus.111401

**Published:** 2026-06-23

**Authors:** Tauram Javed, Mehwish Shafique, Huma Saleem, Almas Iqbal, Asma Akram

**Affiliations:** 1 Anesthesia and Pain Management, Sahiwal Medical College, Lahore, PAK; 2 Anesthesia and Pain Management, Shaukat Khanum Memorial Cancer Hospital and Research Centre, Lahore, PAK

**Keywords:** adrenalectomy, bilateral pheochromocytoma, catecholamine secreting tumor, endocrine anesthesia, men2a, parathyroidectomy, secretory tumors of adrenal gland, thyroidectomy

## Abstract

This is a classic and complete representation of the multiple endocrine neoplasia type 2A (MEN2A) syndrome, involving primary hyperparathyroidism, medullary thyroid carcinoma, and pheochromocytomas. This case is particularly noteworthy for the recurrent, contralateral presentation of the pheochromocytoma, which emerged years after the initial surgical management. Recurrent and contralateral pheochromocytomas present unique anesthetic, surgical, and postoperative problems due to excess catecholamines and severe, potentially life-threatening hemodynamic instability in the perioperative phase. We report an unusual case of a 29-year-old female with MEN2A who had a right adrenalectomy, subsequently total thyroidectomy and parathyroidectomy, and was under follow-up with an endocrinologist. During follow-up, she was found to have very high plasma normetanephrine levels (>760 pg/mL), and radiological imaging revealed an avid adrenal nodule in the left adrenal gland, which was consistent with the diagnosis of recurrent contralateral pheochromocytoma. She was managed preoperatively on doxazosin and metoprolol. During the anesthetic evaluation, she had orthostatic hypotension, but no other symptoms related to pheochromocytomas. Elective open left adrenalectomy was carried out under general anesthesia, aiming to blunt stress responses. Hemodynamics were monitored invasively and managed with vasodilator and vasopressor infusions. After the removal of the adrenal tumor, the noradrenaline infusion was stopped as she maintained her stable hemodynamic parameters. She had an uneventful recovery and an orderly discharge from the post-anesthesia recovery unit. This case highlights the importance of meticulous preoperative optimization, vigilant intraoperative monitoring, and readiness with vasoactive agents in managing pheochromocytomas in patients with MEN2A. A multidisciplinary approach is essential to achieve a safe anesthetic course and a favorable outcome in such high-risk scenarios.

## Introduction

Multiple endocrine neoplasia type 2A (MEN2A) is an autosomal dominant disorder most commonly characterized by medullary thyroid carcinoma, pheochromocytoma, and, less frequently, primary hyperparathyroidism. While pheochromocytoma is a well-recognized surgical challenge due to catecholamine surges and cardiovascular instability, most available literature focuses on the primary resections, and managing a metachronous contralateral recurrence presents unique anesthetic challenges [1]. Recurrent or contralateral pheochromocytoma in MEN2A is less frequently discussed, despite its additional complexities [2]. Altered physiology, a depleted catecholamine buffer capacity from prior surgeries, and lifelong endocrine surveillance make anesthetic management more demanding. This case highlights perioperative management strategies in a patient with MEN2A presenting with recurrent pheochromocytoma after right adrenalectomy and thyroidectomy [3]. This tumor is rare in the Pakistani population, but its incidence has not yet been reported. The greatest frequency occurs in the fourth and fifth decades of life, with a slightly higher female preponderance. There is a gap in the national literature regarding the anesthetic implications of managing MEN2A and pheochromocytoma in Pakistan [4].

## Case presentation

A 29-year-old woman with a known diagnosis of MEN2A underwent right adrenalectomy and total thyroidectomy with parathyroidectomy in 2022 for pheochromocytoma and medullary thyroid carcinoma. For the past two and a half years, the patient has remained clinically stable, maintained on levothyroxine 150 mcg daily and vitamin D supplementation as a part of her replacement therapy. During routine follow-up in 2024, clinically asymptomatic was found to have markedly elevated plasma normetanephrines (Table 1) [5], and CT imaging revealed a left adrenal nodule. Functional imaging with DOTA-PET [1] confirmed a solitary, avid lesion with no evidence of metastasis, consistent with recurrent pheochromocytoma. The endocrinologist optimized her treatment by starting her on an alpha and beta blocker preoperatively. She continued doxazosin 10 mg twice daily for alpha-blockade and metoprolol 25 mg once daily for beta-blockade.

**Table 1:** Biochemical results. NA: not available; ND: not detected; PTH: parathyroid hormone

Normal values	Baseline values (September 2021)	Post-right adrenalectomy (January 2022)	Post-thyroidectomy (March 2022)	Disease recurrence (October 2024)	Post-left adrenalectomy (February 2025)
Metanephrine (less than 90)	>650 pg/mL	16.79 pg/mL	25.65 pg/mL	10.948 pg/mL	8.448 pg/mL
Normetanephrine (less than 190)	>900 pg/mL	112.2 pg/mL	104.7 pg/mL	>760 pg/mL	60.57 pg/mL
Calcitonin (ND: 5.0)	5.86 pg/mL	5.89 pg/mL	9.39 pg/mL	<1.89 pg/mL	<1.89 pg/mL
Calcium (8.5-10.5)	10.61 mg/dL	11.46 mg/dL	9 mg/dL	9.7 mg/dL	9.7 mg/dL
PTH (18.5-88)	95.1 pg/mL	49 pg/mL	<4.6 pg/mL	NA	NA
TSH (0.35-5.5)	1.91 µIU/mL	0.96 µIU/mL	38.9 µIU/mL	4.313 µIU/mL	1.16 µIU/mL

A computed tomography (CT) scan for an adrenal protocol was performed, which confirmed the presence of a lesion measuring 11 × 15 mm in the left adrenal gland and evidence of a previously performed right adrenalectomy (Figure 1). The nodule showed no internal calcification or hemorrhage.

**Figure 1:**
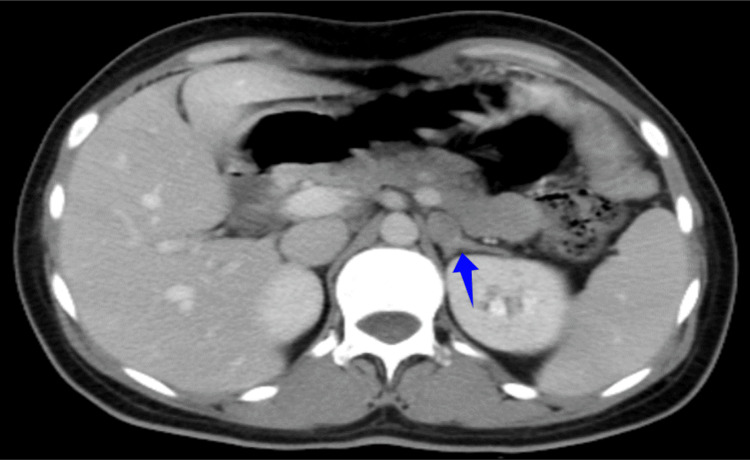
Preoperative adrenal protocol CT showing a well-circumscribed left adrenal nodule (blue arrow) measuring 15 × 11 mm, with a prior right-sided adrenalectomy.

The patient was evaluated in the anesthesia pre-operative clinic for a scheduled open left adrenalectomy. She had a known diagnosis of MEN2A with successful previous surgeries. Despite being normotensive at that time, her previous anesthesia history was notable as she experienced a severe hypertensive episode at induction that led to cancellation of the procedure. An echocardiogram revealed a normal ejection fraction of 55% with no abnormalities in left ventricular wall motion or function. She was continued on doxazosin and metoprolol, with well-controlled blood pressure and heart rate.

The anesthesia assessment before surgery, using Roizen’s criteria [6], yielded a score of 1 out of 4, with a positive finding of orthostatic hypotension. Blood pressure dropped significantly from 110/70 mmHg in the lying position to 60/40 mmHg upon standing. The ECG showed sinus arrhythmia with irregular R-R intervals. This echocardiogram showed normal ejection fraction and good biventricular function. The airway assessment did not anticipate any difficulty with the airway, given a Mallampati class I [6], adequate mouth opening, a thyromental distance of more than 6 cm, and good neck extension. ASA physical status was assessed as class II [7]. Antihypertensive medications were not altered based on the patient's prior medical history.

On the morning of surgery, her prescribed medications were continued. Standard ASA monitoring [7] was instituted along with invasive arterial and central venous access. Pre-induction vitals were stable (BP 104/57 mmHg, HR 71 bpm, SpO₂ 98%). The arterial line was inserted under local anesthesia before induction. Anesthesia was induced with fentanyl, midazolam, propofol, and atracurium, and the trachea was intubated. Ventilation was volume-controlled with sevoflurane for maintenance; 2 g of magnesium sulfate and 100 mg hydrocortisone were administered at induction. Given her risk profile, vasoactive and antihypertensive medications were prepared before incision. Noradrenaline infusion was initiated at a low dose and titrated according to hemodynamic requirements, along with IV fluids. Interestingly, no hypertensive crises occurred during tumor handling, and hypotension was managed smoothly with noradrenaline support. Following adrenalectomy, the infusion was tapered and discontinued.

At the end of the procedure, she received epidural analgesia with bupivacaine at 0.125% concentration, administered at a rate of 10 mL per hour for post-op pain relief. Neuromuscular blockade was reversed, and the patient was extubated in the operating room. She was then transferred to the post-anesthesia care unit with stable vitals and subsequently recovered without complications.

## Discussion

This case highlights that perioperative management of MEN2A-associated pheochromocytoma requires an individualized, multidisciplinary approach. The unique risks of MEN2A, such as bilateral or recurrent tumors and previous endocrine surgery, require lifelong follow-up and prompt detection of recurrence. MEN2A is closely linked with bilateral adrenal involvement, and recurrence following unilateral adrenalectomy is well described [2]. Early diagnosis of our patient through biochemical screening, followed by functional imaging, facilitated the early planning of definitive surgery. In contrast to sporadic pheochromocytoma [8], MEN2A-related recurrences demand meticulous attention to prior surgical and hormonal manipulation, which may reduce physiological reserve and make perioperative management challenging.

Pheochromocytoma is one of the most physiologically demanding tumors to encounter in anesthesia because of its unpredictable secretion of catecholamine. Sudden intraoperative hypertension, arrhythmias, and postoperative hypotension are familiar risks. Early pheochromocytoma surgery has a 45% mortality and intraoperative complications [9]. In MEN2A, these are compounded by lifetime endocrine disturbances and the risk of recurrent or contralateral disease. In our patient who had a right adrenalectomy in the past, the appearance of a left adrenal lesion shows a classic metachronous presentation. This highlights the need for lifelong monitoring with plasma or urinary metanephrine tests and regular imaging [10].

Alpha blockade is a standard practice and requires modification for optimum control of blood pressure and restoration of blood volume. Therefore, preoperative management involves initiating alpha-blockers (e.g., phenoxybenzamine, doxazosin) to control hypertension [11]. Selective β₁-blockers (e.g., propranolol, metoprolol) are also used to control tachyarrhythmias that may result from epinephrine or dopamine-secreting tumors; however, these must be initiated after complete alpha blockade. This avoids unopposed alpha-mediated vasoconstriction that could occur after antagonism of β2-mediated vasodilatation, which may precipitate a hypertensive crisis [12]. Importantly, alpha-blockade must precede beta-blockade to avoid unopposed alpha-adrenergic stimulation, which can precipitate hypertensive crisis [13]. In this case, the patient was on metoprolol tartrate 25 mg once daily in the morning and doxazosin 10 mg at bedtime for control.

Preoperative optimization is crucial in reducing perioperative morbidity and mortality. The Roizen criteria, proposed in 1982, remain widely used to evaluate the adequacy of preoperative preparation [6]. These include no in-hospital blood pressure >160/90 mmHg in the 24 hours before surgery, no orthostatic hypotension with systolic BP <80 mmHg or diastolic <45 mmHg, no ST- or T-wave changes on ECG for one week prior, and no more than five premature ventricular contractions per minute.

Based on these criteria [6], our patient met only one out of four, highlighting the high-risk nature of this case and the need for careful intraoperative management. Nonetheless, our patient manifested substantial orthostatic hypotension, indicating the precarious balance between proper adrenergic control and iatrogenic hypotension [6]. This demonstrates the challenge of titrating medical therapy with the contralateral adrenal already resected, lowering catecholamine buffer capacity.

Patients are often given anxiolytic drugs like midazolam before surgery for anxiolysis. It is best to place an arterial line before induction to closely monitor hemodynamic changes during induction. An epidural is sometimes added to help with pain [14]. For open procedures, pain relief is essential. An epidural helps with pain control perioperatively. Reliable intravenous access during surgery is necessary, ideally with two good peripheral IV lines, so that medications and fluids can be handled safely.

For individuals with inadequate left ventricular function, a central venous access may be inserted for the administration of vasopressors or inotropes. Patients with cardiomyopathy would require narcotic-based induction or cardio-stable gradual induction with divided doses of fentanyl, morphine, propofol, and cisatracurium. Because ketamine has sympathomimetic effects, it should be avoided. A smooth induction of anesthesia with an ideal depth of anesthesia is the primary objective in order to prevent sympathetic surge during endotracheal intubation and laryngoscopy.

Patients with MEN2A are typically associated with tachycardia and hypertension [15], which may be exacerbated in pheochromocytoma because of catecholamine release. Patients may experience ischemia and arrhythmias as a result of severe hypertension and tachycardia. Sevoflurane has hemodynamic qualities and a lack of arrhythmogenic potential, and is the most often utilized inhalational drug for maintaining anesthesia during pheochromocytoma resection. Avoid sympathomimetics (e.g., ephedrine, ketamine) and histamine-releasing drugs (e.g., atracurium, morphine). Metoclopramide is contraindicated because it can cause adrenergic myocarditis, cardiogenic shock, and hypertensive crisis.

Intraoperative administration of magnesium sulfate was utilized [16], since it offers vasodilatory and anti-arrhythmic effects, which can potentially blunt surges in catecholamines. Adjunctive measures are frequently under-reported in recurrent pheochromocytoma but could prove to be very useful [3]. The primary anesthetic issues were hemodynamic instability upon tumor manipulation. Steroid replacement and hypotension following resection. While our patient stabilized, on IV fluids and vasoactive agents (noradrenaline). The studies showed that preparedness and anticipation were just as crucial as the intervention itself [3,17]. With previous adrenalectomy, perioperative glucocorticoid supplementation was necessary to avoid adrenal insufficiency. This forgotten detail is crucial in recurrent or bilateral adrenal disease. Following tumor resection, the removal of catecholamine excess usually results in severe hypotension.

This case shows that recurrence of pheochromocytoma in MEN2A is not a mere recurrence of the initial presentation. Rather, it presents with special anesthetic challenges as prior altered baseline physiology secondary to prior endocrine surgery, enhanced risk of intraoperative hypotension with preoperative blockade, steroid supplementation for life, and psychological and logistical challenges of multiple interventions.

Multidisciplinary collaboration among endocrinologists, surgeons, and anesthesiologists was critical to the successful outcome. The case validates current recommendations for lifelong biochemical monitoring in MEN2A and highlights the importance of anesthetic caution even in clinically stable-appearing patients preoperatively.

Successful management in this case was not the result of pharmacologic optimization alone but of coordinated input from the multidisciplinary team (MDT), particularly endocrinology and anesthesia. Close endocrine anesthesia collaboration allowed careful titration of alpha and beta blockade, fluid management, and intraoperative hemodynamic control. This highlights that in pheochromocytoma, structured MDT involvement is as important as anesthetic technique itself, since endocrine optimization directly determines anesthetic safety [17,18]. The lessons from this case reinforce the need for clear preoperative planning, shared decision-making, and postoperative coordination among specialties to improve patient outcomes in MEN2A.

The limitations in this report are the experience of a single patient. Although it brings out useful practical strategies and pitfalls, the result is not generalizable. More evidence from case series or registries is required to more clearly define best practice in anesthetic management of recurrent pheochromocytoma in MEN2A [17,18].

## Conclusions

This case highlights the fact that perioperative management of MEN2A-associated pheochromocytoma requires an individualized, multidisciplinary approach. The unique risks of MEN2A, such as bilateral or recurrent pheochromocytomas and previous endocrine surgery, require lifelong follow-up and prompt detection of recurrence. Preoperative management with carefully titrated alpha- and beta-blockage is necessary, but even normotensive patients can exhibit unpredictable instability, highlighting the importance of careful assessment against Roizen's criteria and judicious management of fluid and drugs. Intraoperatively, invasive monitoring, anticipation of rapid hemodynamic changes, and preparedness with vasoactive drugs and steroid supplementation are essential for safe anesthesia. Most importantly, systematic collaboration among endocrinology, anesthesia, and surgical teams facilitates synchronized planning, adaptability during crises, and maximized outcomes.
